# Factors associated with medical student clinical reasoning and evidence based medicine practice

**DOI:** 10.5116/ijme.563a.5dd0

**Published:** 2015-11-08

**Authors:** Arpana R. Vidyarthi, Robert Kamei, Kenneth Chan, Sok-Hong Goh, Lek Ngee

**Affiliations:** 1 Duke-NUS (National University of Singapore) Graduate Medical School, Singapore

**Keywords:** Medical student education, evidence based medicine, clinical reasoning, curriculum development, clinical culture

## Abstract

**Objectives:**

To identify the factors associated with medical students’ clinical reasoning (CR) use and evidence-based medicine (EBM) use in the clinical setting.

**Methods:**

Our cross-sectional study surveyed 44 final-year medical students at an emerging academic medical center in Singapore. We queried the students’ EBM and CR value and experiences in the classroom and clinical settings. We compared this to their perceptions of supervisors’ value and experiences using t-tests. We developed measures of teaching culture and practice culture by combining relevant questions into summary scores. Multivariate linear regression models were applied to identify factors associated with the students’ CR and EBM clinical use.

**Results:**

Eighty-nine percent of students responded (n=39). Students reported valuing CR (p=0.03) and EBM (p=0.001) more than their supervisors, but practiced these skills similarly (p=0.83; p=0.82). Clinical practice culture and classroom CR experience were independently associated with students’ CR clinical use (p=0.05; p=0.04), and classroom EBM experience was independently associated with students’ EBM clinical use (p=0.03). Clinical teaching culture was not associated with students’ CR and EBM clinical use.

**Conclusions:**

Our study found that medical students’ classroom experience and the clinical practice culture influenced their CR and EBM use. The clinical teaching culture did not. These findings suggest that in order to increase student CR and EBM use, in addition to providing classroom experience, medical educators may need to change the hospital culture by encouraging supervisors to use these skills in their clinical practice.

## Introduction

Clinical Reasoning (CR) is a structured way in which seasoned physicians make diagnoses. It comprises several skills which include applying pattern recognition, problem representation, context formulation, differential diagnosis, comparing/contrasting, and hypothesis generation.^1^ Evidence-Based Medicine (EBM) is the process of finding, applying and evaluating evidence to make clinical decisions.^2^ These two skills are the cornerstones of exemplary clinical practice and doctors use them to determine accurate diagnoses,^3^ initiate best-practice treatment plans,^2^ and keep patients free from medical errors.^4^ Although CR and EBM are considered central to physician’s competence,^5^ they are not used as frequently as they ought to be worldwide.^6^ As many physician practice habits are established during their medical school years,^7^ understanding the factors associated with medical student CR and EBM use may be foundational to increasing the application of these skills to current and future patient care.

In recognition, many medical schools have developed curricula to teach these skills. Published curricular evaluations have shown an increase in student CR and EBM knowledge and skills.^8^^-^^11^ Most evaluations assessed students in the classroom or simulated settings, and few investigated students’ CR and EBM use in actual clinical settings.^12^ Although these curricula may influence student clinical practice, to our knowledge, no study has explored whether these curricula, or other factors, are associated with student CR and EBM application to patient care in the clinical setting.

One of these factors may be the culture in which the students receive their clinical training (clinical culture). Although “culture” has many definitions, key components include the values and norms of the environment.^13^

Clinical culture is set by clinical supervisors’ values and behaviors, which in turn establishes the clinical norms. These norms are transmitted to students through the informal and hidden curriculum, which can influence their behaviors.^14^

Medical students today receive their clinical training in a variety of settings. Traditionally, university, public, or government-based academic settings were the main training grounds, but with increasing frequency, private and community hospitals also participate in medical student training. Each of these settings has a distinctive clinical culture. Globalized medical education brings western medical schools together with local hospitals in various countries transforming existing hospital systems into teaching or academic settings. Given these changes to the student training landscape, medical students may experience clinical cultures that differ from their medical school culture. The impact of these differences on medical student clinical practices may be best elucidated in a setting where the school and its associated hospital have vastly different cultures. One such setting is an emerging academic medical center, where a medical school and established hospital are in the process of integrating.

In this study, we aimed to explore the relationships between the classroom experience, the clinical culture, and student clinical practices at an emerging academic medical center in Singapore. We developed measures of classroom experiences, clinical teaching culture, and clinical practice culture, and hypothesized that aspects of the clinical culture would be associated with our medical students’ CR and EBM clinical use.

## Methods

### Site and participants

Duke-NUS Graduate Medical School (Duke-NUS), a 4-year US-model medical school, was established in 2007. It matriculates approximately 44 students yearly who hold an undergraduate degree. In 2012, Duke-NUS and Singhealth, the hospital in which the medical students receive their clinical training, formally united to form an academic medical center. The National University of Singapore Committee on Human Research approved the study.

### Survey development

As no existing instrument was validated to evaluate our study aims, we developed a survey that queried student perceptions and experiences with CR and EBM. We drew from the Theory of Reasoned Action^15^ and theories of Organizational Culture^16^ as the foundation of our instrument. As self-reports may yield inaccurate estimates of competence,^17^ our survey queried students’ perceptions of their values and use of CR and EBM directly and through hypothetical scenarios. Survey questions were created by experts in medical education and psychometrics, and piloted by medical school faculty to establish content and face validity. As a check of usability and language, clinical faculty and non-participant medical students piloted the instrument, after which items were re-worded, reformatted, or eliminated.

Most items asked for students to rate their level of agreement on a 5-point Likert scale. (1=strongly disagree, 5=strongly agree) A subset of questions asked students to rate the likelihood of a specified behavior on a 5-point scale (1=very unlikely, 5=very likely). The Cronbach's alpha for the survey in its entirety was 0.87. The full survey is included in Appendix 1.

### Survey measures

All survey measures were rated on a 5-point Likert agreement scale unless otherwise specified.

### Student CR/EBM value and practice behaviors

We asked students to rate their agreement with statements on the value of CR and EBM. We also asked them to report their CR and EBM practice or use during the course and on their most recent rotation.

### Supervisor CR/EBM value and practice behaviors

We asked students to report their agreement with statements on how their clinical supervisors’ value and use CR and EBM in their clinical practice.

### Clinical environment overall

To understand the clinical environment (in which faculty teach, practice, and supervise while students practice and learn) we posed two sets of questions. The first asked students to rate their agreement with statements regarding their supervisor’s CR and EBM practice and teaching. The second set of questions asked students to reflect on a clinical scenario and asked about the supervisors likelihood (rated on a 5-point likelihood scale described above) to transparently reason through the case, use the medical literature to inform decision making, and appreciate the student’s use of EBM to inform the case.

### Clinical and teaching culture: summary scores

We characterized the clinical culture by identifying questions that explored the CR/EBM norms (i.e., supervisor behaviors) and values (i.e., perception of supervisor values) in the realms of clinical practice and teaching. We chose items that asked students perceptions directly from their experience as well as those from the theoretical scenarios to increase validity of the responses and categorized them into “practice culture” or “teaching culture”. Those items that demonstrated high internal consistency when combined were termed the “practice culture summary score” or “teaching culture summary score” respectively.

### Procedures and data analysis

The survey was distributed electronically to all final year medical students at Duke-NUS immediately upon completion of a CR /EBM course. Participation was voluntary and consent was implied with the return of the survey. Responses were collected anonymously for 6 weeks during which 3 reminders were sent via email. We analyzed the survey by characterizing the distribution of students’ responses using univariate statistics (denoting a value of ≥ 3.75 as “agreement”), and evaluated bivariate associations using Student’s t-tests. Multivariate linear regression models identified factors associated with student CR and EBM clinical use. We selected variables for entry into models based on our a-priori hypothesis of the factors that would influence student clinical CR/EBM use and included the practice culture summary score, the teaching culture summary score, and classroom experience of EBM or CR. We designated p ≤ 0.05 to be statistically significant. All analyses were performed using SAS version 8.12 (SAS Institute Inc, Cary, NC.).

## Results

Forty-four students were eligible for the study and 39 (89%) responded to the survey. The class as a whole was gender balanced (55% female/45% male), diverse (4 ethnicities declared), and represented a wide range of clinical interests (15 specialties for subsequent residency training). Full demographics of the class as a whole are found in Table 1.

**Table 1:** Characteristics of the Duke-NUS graduate medical school class of 2013 (N=44)

Characteristics		Number of Students	%
Age			
	<25	1	2
	25-29.9	31	7
	30-34.9	12	27
Gender			
	Male	20	45
	Female	24	55
Ethnicity			
	Chinese	33	75
	Other Asian	5	11
	Caucasian	3	7
	Indians	2	5
	Malay	1	2
Post Medical School Training Program			
	Medical Officer	10	23
	Internal Medicine	6	13
	Pediatric Medicine	6	13
	Anesthesia	4	9
	Orthopedic Surgery	3	7
	Ophthalmology	2	5
	Psychiatry	2	5
	Emergency Medicine	2	5
	General Surgery	2	5
	Radiology	2	5
	Cardiothoracic Surgery	1	2
	Family Medicine	1	2
	Obstetrics and Gynecology	1	2
	Pathology	1	2
	Plastic Surgery	1	2

### Comparison of student and supervisor CR/EBM value and clinical practice

Students’ reports of value were higher than their perceptions of supervisors value in CR (student mean 4.23 [SD 0.75], supervisor mean 3.90 [SD 0.74]; p=0.03) and EBM (student mean 4.18 [SD 0.70], supervisor mean 3.68 [SD 0.83]; p=0.001); however, their CR and EBM clinical use were similar to their perceptions of supervisor practice in these domains (CR practice: student mean 3.78 [SD0.73], supervisor mean 3.95 [SD 0.64], p=0.83; EBM practice: student mean 3.35 [SD 0.77], supervisor mean 3.50 [SD 0.93], p=0.82) These data are presented in Table 2.

**Table 2:** Duke-NUS student perceptions: student and supervisor clinical reasoning and evidence based medicine value and clinical use

Student Perceptions	Clinical Reasoning			Evidence Based Medicine		
	Student Mean (SD)	SupervisorMean (SD)	*P*value	Student Mean (SD)	Supervisor Mean (SD)	*P*value
Value	4.23 (0.75)	3.90 (0.74)	0.03	4.18 (0.7)	3.68 (0.83)	0.001
Clinical use	3.78 (0.73)	3.95 (0.64)	0.83	3.35 (0.77)	3.50 (0.93)	0.82

### Practice culture summary score

We identified 5 items in the survey that represented the clinical practice culture: agreement with statements on supervisor value and use of CR and EBM (4 items) and the likelihood that the supervisor would look to the medical literature to inform clinical decision making in a theoretical scenario (mean 2.98 [SD 1.00]; combined mean 3.60; cronbach α= 0.91; eigenvalue 3.72).

### Teaching culture summary score

We identified four items that represented clinical teaching culture: agreement with supervisor teaching CR (mean 3.63[SD 0.98]), agreement with supervisor EBM (mean 3.28 [SD 1.09]), transparency in CR (mean 2.90 [SD 1.15]), and appreciation of the student’s use of these skills (mean 3.45 [SD 0.75]) (combined mean 3.31; cronbach α=0.90; eigenvalue 3.16).

### Factors contributing to student CR and EBM clinical use

In a multivariable model (that included CR practice/experience in the classroom, teaching culture and practice culture summary scores), the practice culture summary score and practicing CR in the classroom were independently associated with students CR clinical use (p=0.05 and p=0.04 respectively). In a model that included EBM classroom practice and the aforementioned summary scores, only practicing EBM in class was independently associated with student EBM clinical use (p=0.03). Multivariable model results with the specific components of the summary scores are depicted in Table 3.

**Table 3:** Factors associated with student clinical reasoning and evidence-based medicine use in the clinical setting

Factors	Students’ Clinical Reasoning Use in theClinical Setting		Students’ Evidence-basedMedicine Use in the Clinical Setting	
	Parameter estimate	*P *value	Parameter estimate	*P* value
Teaching culture summary score *composite of 4 questions representing the teaching culture: supervisor teaching CR/EBM, transparency of CR, and appreciation students skills in these (crohnbach α=0.90, eigenvalue 3.72; mean 3.60)	0.09	0.61	0.17	0.40
Practice culture summary score *composite of 5 questions representing practice culture: supervisor value and use of CR/EBM, and likelihood of using the medical literature to inform decision making. (crohnbach α=0.91 eigenvalue 3.16; mean 3.31)	0.46	0.05	0.33	0.21
Classroom experience with the skill	0.27	0.04	0.32	0.03

## Discussion

In this cross-sectional study of students at an emerging academic medical center, we found that our medical students valued CR and EBM more than their supervisors, but clinically used these skills similarly. We also found that classroom experience and clinical culture, set by supervisors’ values and clinical practices, was associated with student CR use; however, clinical teaching was not associated.

Our preliminary study is the first to explore the potential roles of classroom teaching and clinical environment on student CR and EBM practice and offers a glimpse into the challenges faced by medical educators worldwide to establish sound clinical behaviors for their students. The overarching construct of culture is useful to consider each of our findings. We detected differences in CR and EBM values between our students and their perceptions of faculty supervisors. These values are likely inculcated by the organizational culture,^16^ specifically the medical school for students and hospital for faculty. These differences may spark a healthy debate thereby adding to the vibrancy of an established academic medical center. However, in an emerging one, it may cause a cultural clash that is disruptive and detracting from the intended milieu. These cultural differences may need to be transparently addressed to promote the positive environment for students to learn and thrive.

Although we found differences between our students’ perceptions of their values and their supervisors’ values, their reported clinical practices were similar. This discordance is informed by theory of reasoned action, which posits that behaviors are a function of subjective norms, evaluation of behavior, as well as an individual’s beliefs.^15^ These subjective norms and evaluations are established by clinical faculty/supervisors and transmitted to the students through the hidden curriculum, a set of influences that function at the level of organizational structure and culture.^18^ The hidden curriculum has previously been reported to influence student attitudes,^19^^-^^21^ and our findings reveal its additional power to influence student clinical behaviors. We further delineated clinical culture into two components, the teaching culture and practice culture. We found that the practice culture, and not teaching culture, was associated with student CR clinical use. This affirms the clinical practice of supervisor’s role in the hidden curriculum and its association with student clinical practice. Although we did not detect this association with student EBM use, we believe that this could be due to the large gap between supervisor and student EBM values or a limitation in our power to detect such a difference. Medical schools and hospitals invest time, energy, and resources into training faculty to teach in the clinical setting. Our findings suggest that investments targeting faculty clinical practices might offer higher returns. To be effective, these efforts should include a comprehensive approach targeting individuals and the system, thereby bringing about a culture change.^22^ For the individual faculty, strategies to change their clinical practice may include developing their CR and EBM self-efficacy and assisting them to overcome previous practice inertia.^23^ By developing faculty’s EBM and CR clinical practice, we can align the formal curriculum, delivered through a CR/EBM course, with the hidden and informal curriculum thereby enhancing medical student learning.

Our formal curriculum, the CR/EBM course, was also associated with student CR and EBM clinical use. Although debate exists on the value of classroom teaching, we believe that the curriculum we developed added value not only due to its pedagogy and content but also due to the consistent hidden curriculum. By inviting respected clinical faculty to teach and protecting student’s time to attend the course while on clinical rotations, we conveyed high value and importance of CR and EBM, which may have contributed to our findings.^1^

Our study had several limitations. Our survey relied on student retrospective self-reports. To reduce the impact of potential self-report bias, we queried student’s values and use as opposed to perceived competence. Also, as student perceptions of supervisors reflect their experience of the culture, their perceptions are germane. In addition, our study had a small number of students. We believe that our timely data collection and high response rate lends to the potency of our findings. While the small sample size may call into question the generalizability of our results, the sample size was sufficiently large enough to allow for a multivariable analytic approach. This approach allowed for greater insight into the relationships between these complex constructs. We conducted our study at a single site in Singapore, which may also limit generalizability. As our organization is undergoing a transition into an academic medical center, this setting might unveil findings that are difficult to elucidate, even if present, in other settings. Furthermore, our students are ethnically diverse and represent a variety of specialty choices, which may make our setting relevant in the face of increasing diversity and globalization of medical student populations. Further research including independent evaluation of clinical practice and ethnographic descriptions of the clinical culture will further be necessary to deepen our understanding of these domains.

## Conclusions

CR and EBM are essential skills for medical students to learn and use. Although many schools offer classroom curricula, the impact of these curricula may not be fully realized without addressing the culture where the students practice and train clinically. This culture exerts powerful influence on medical student clinical practice through the hidden curriculum. As cultures are neither pure nor static, medical schools and the variety of hospital systems that now train students can come together to align classroom teaching with clinical practice, thus creating a culture that promotes effective student learning. In this way, medical schools and hospitals together may advance reasoned and evidence-based care today and into the future.

### Acknowledgements

Gurpreet Dhaliwal, Professor, University of California San Francisco, for assistance in study design and critical review of the manuscript. Patti Katz, Professor, Institute of Health Policy Studies, University of California, for her assistance in study and instrument design. Scott Compton, Associate Professor, Duke-NUS Graduate Medical School for his contributions to the design of the study and analysis.

### Conflict of Interest

The authors declare that they have no conflict of interest.

## Supplementary material

Supplementary file 1 Clinical reasoning and evidence-based medicine experiences survey
