# Supplementary material for: Factors associated with medical student clinical reasoning and evidence based medicine practice
Source: Int J Med Educ. 2015 Nov 8;6:142–8. doi: 10.5116/ijme.563a.5dd0 (PMC4646359; doi:10.5116/ijme.563a.5dd0)
Supplement: Supplementary file 1 — Clinical reasoning and evidence-based medicine experiences survey [file ijme-6-142-S1.pdf]

Appendix 1

Clinical Reasoning and Evidence-Based Medicine Experiences Survey

Dear Student,
We would like to understand your Singapore clinical learning environment. This information will help us refine our courses to better meet your needs.
Thank you for taking the survey and providing us with your input regarding the questions.

1. Think about your experience during Practice Course 4 and rate the extent to which you agree with the following:

Item	1	2	3	4	5
I actively thought through clinical cases presented in class.	❍	❍	❍	❍	❍
I was exposed to different systematic approaches to reasoning through clinical cases in class.	❍	❍	❍	❍	❍
I practiced using systematic approaches to reasoning through cases in class.	❍	❍	❍	❍	❍
I practiced using the current medical literature to answer clinical questions that arose during the class.	❍	❍	❍	❍	❍

2. Think about your experiences in your Fourth Year of medical school while on Singapore clinical rotations. Please rate the extent to which you agree with the following:

Item	1	2	3	4	5
I reasoned through cases more effectively.	❍	❍	❍	❍	❍
I took better care of patients.	❍	❍	❍	❍	❍
I used the current medical literature to answer clinical questions that arose for my patients.	❍	❍	❍	❍	❍

3. Think about your most common experiences in Singapore for your 4th year clinical rotations.

Please rate the extent to which you agree with the following:

Item	1	2	3	4	5
I used a systematic approach to reason through my patients' case.	❍	❍	❍	❍	❍
I used the current medical literature to answer clinical questions that arose for my patients.	❍	❍	❍	❍	❍

4. Please rate the extent to which you agree with the following:

Item	1	2	3	4	5
I value systematic approach to reasoning for exemplary patient care.	❍	❍	❍	❍	❍
I value using the current medical literature to answer clinical questions for exemplary patient care.	❍	❍	❍	❍	❍
This course adds value to my clinical training.	❍	❍	❍	❍	❍

5. Think about your most common 4th year clinical rotations in Singapore and the consultant supervisors who were in charge of your clinical educational experience and patient care. From your perspective, please rate the extent to which you agree with the following:

Item	1	2	3	4	5
Uses the current medical literature to answer clinical questions that arise for patients.	❍	❍	❍	❍	❍
Uses a systematic approach to reason through patients' cases.	❍	❍	❍	❍	❍
Values using a systematic approach to reasoning through cases for exemplary patient care.	❍	❍	❍	❍	❍
Values using the current medical literature to make decisions for exemplary patient care.	❍	❍	❍	❍	❍
Teaches me how to use the current medical literature to answer clinical questions.	❍	❍	❍	❍	❍
Teaches me how to systematically reason through patients' cases.	❍	❍	❍	❍	❍

6. Scenario

You are on a clinical rotation in Singapore. Think of your most common experience.

You are caring for a patient with your team members whose treatment plan is under discussion.

Ultimately, the consultant makes a decision on the treatment plan.

Item	1	2	3	4	5
How likely is it that the consultant will fully explain his/her thinking process for this decision?	❍	❍	❍	❍	❍
How likely is it that the consultant will look to the current medical literature to inform this decision?	❍	❍	❍	❍	❍

7. You do a literature search and find an article that could inform the treatment decision.

Item	1	2	3	4	5
How likely is it that this will be appreciated by the consultant?	❍	❍	❍	❍	❍
How likely is it that this will be appreciated by your resident/medical officer?	❍	❍	❍	❍	❍
How likely is it that this will be appreciated by your intern/house officer?	❍	❍	❍	❍	❍
